# Thermodynamics of Tower-Block Infernos: Effects of Water on Aluminum Fires

**DOI:** 10.3390/e22010014

**Published:** 2019-12-20

**Authors:** John F. Maguire, Leslie V. Woodcock

**Affiliations:** 1 Scientific Simulation Systems Inc., San Antonio, TX 78249, USA; 2 Department Physics, University of Algarve, 8005-139 Faro, Portugal

**Keywords:** combustion thermodynamics, aluminum fire, water extinguisher, Grenfell Tower, tower-block safety

## Abstract

We review the thermodynamics of combustion reactions involved in aluminum fires in the light of the spate of recent high-profile tower-block disasters, such as the Grenfell fire in London 2017, the Dubai fires between 2010 and 2016, and the fires and explosions that resulted in the 9/11 collapse of the World Trade Center twin towers in New York. These fires are class B, i.e., burning metallic materials, yet water was applied in all cases as an extinguisher. Here, we highlight the scientific thermochemical reasons why water should never be used on aluminum fires, not least because a mixture of aluminum and water is a highly exothermic fuel. When the plastic materials initially catch fire and burn with limited oxygen (O_2_ in air) to carbon (C), which is seen as an aerosol (black smoke) and black residue, the heat of the reaction melts the aluminum (Al) and increases its fluidity and volatility. Hence, this process also increases its reactivity, whence it rapidly reacts with the carbon product of polymer combustion to form aluminum carbide (Al_4_C_3_). The heat of formation of Al_4_Cl_3_ is so great that it becomes white-hot sparks that are similar to fireworks. At very high temperatures, both molten Al and Al_4_C_3_ aerosol react violently with water to give alumina fine dust aerosol (Al_2_O_3_) + hydrogen (H_2_) gas and methane (CH_4_) gas, respectively, with white smoke and residues. These highly inflammable gases, with low spontaneous combustion temperatures, instantaneously react with the oxygen in the air, accelerating the fire out of control. Adding water to an aluminum fire is similar to adding “rocket fuel” to the existing flames. A CO_2_–foam/powder extinguisher, as deployed in the aircraft industry against aluminum and plastic fires by smothering, is required to contain aluminum fires at an early stage. Automatic sprinkler extinguisher systems should not be installed in tower blocks that are at risk of aluminum fires.

## 1. Introduction

A well-known adage of the advice given in the event of a kitchen fire is, “Never put water on burning liquids!” There are several good reasons for this longstanding rule of engagement in the health and safety of professional fire-fighting literature. In the case of fires involving pans of hot cooking oil, for example, the main reason is that the heavier water with the lower boiling point would immediately vaporize and spray the burning oil all around, thereby spreading the fire out of control. Fires are classified according to the rules for extinguishing them; inflammable liquids and molten plastics or metals are class B [1]. These fires involve liquids such as cooking oil, gasoline, diesel fuel, alcohol, and molten plastics, all of which emit flammable vapors at the liquid surface. It is these vapors that burn, not the liquid. If the surface of the liquid is static, the area is the smallest that it can be. If the liquid is disturbed, it increases the surface area and amounts of flammable vapors released, thus increasing the extent of the fire. That is the main reason never to pour water on stovetop cooking oil fires. Successful class B extinguishment is accomplished by starving the fire of oxygen, i.e., by smothering.

If the fire involves an aluminum pan, there is another good reason not to put water on it. A kitchen fire caused by leaving an aluminum pan of food, which partially burns to carbon, on a hot stove can also lead to a catastrophic conflagration. If the aluminum melts and reacts with the carbon, it becomes aluminum carbide (Al_4_C_3_) which, when dry, is a harmless, yellowish brown powder. However, aluminum carbide reacts with water to give aluminum hydroxide (Al(OH)_3_) plus the well-known natural gas fuel, methane (CH_4_). At high temperatures, the reaction speed increases and the products are alumina (Al_2_O_3_) + CH_4_. The reaction rate becomes fiercely fast, as the highly inflammable methane adds fuel to the flames, releasing more energy to further increase the temperature.

The chemistry of metallic carbides reaction with water is well established. Molten aluminum in contact with hot carbon readily forms aluminum carbide, which reacts with water to give off 3x equimolar amounts of methane (CH_4_) gas. In the case of calcium carbide (Ca_2_C), the gas produced is acetylene (C_2_H_2_), which combines with oxygen (O_2_) in air to produce the hottest known flame, as used in oxyacetylene welding. The controlled application of water to calcium carbide has long been a source of heat or light in the antique carbide lamps. This general chemistry between many metallic carbides and water is one means of storing very high-energy gaseous fuels, such as methane, acetylene, and hydrogen. It was first reported in nature as long ago as 1896 [2].

QUOTE: “The construction of the electric furnace by M. Moissan in 1893, in which the heating power of the electric arc was directly utilized, by extending the upper limit of working temperatures, fused aluminum takes up carbon readily with formation of the crystalline carbide AlC, and the oxides of many other metals furnish similar crystalline compounds when heated in the electric furnace with an excess of carbon. The behavior of these substances with water furnishes the most convenient mode of classification. Of those reacting with water, the carbides of lithium, LiC, calcium, CaC, strontium, SrC, and barium, BaC, furnish pure acetylene; of aluminum, AlC, and of beryllium, BeC, pure methane; of manganese, MnC, a mixture of equal volumes of hydrogen and methane; whilst the metals of the cerite group give crystalline carbides of the type RC (CeC, LaC, YC, and ThC), all of which react with cold water, forming a complicated gas mixture containing hydrogen, acetylene, ethylene, and methane.”

**Figure 1:**
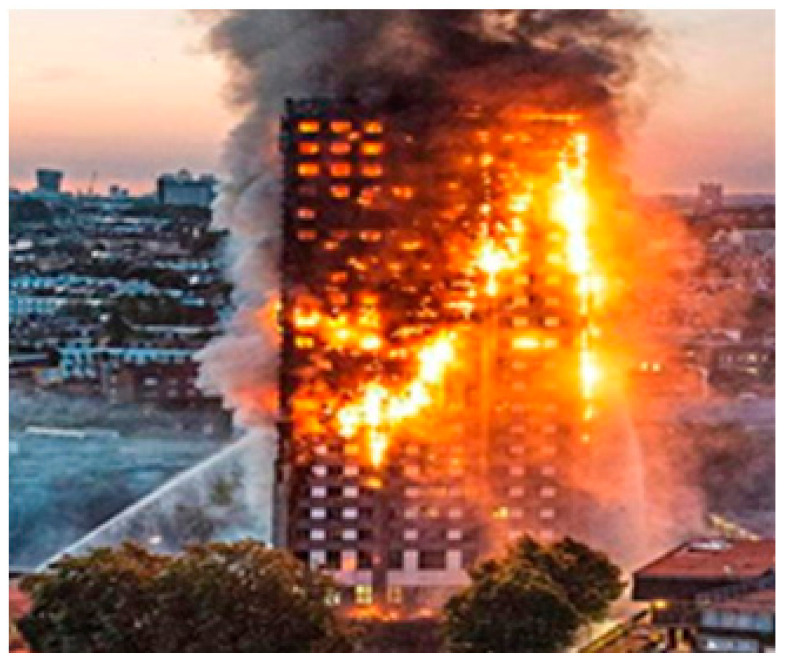
Burning Grenfell Tower at the height of conflagration; the black smoke at the top is combustible material burning without water; the whitish gas is mainly alumina dust from the reaction of Al and or Al_4_C_3_ and steam, as the water still being applied. Note the intensity of the conflagration where the water hits the building, and the associated whitish clouds around.

Thus, we have known for at least 120 years that when the predominant materials are molten aluminum and molten polymers, the combustion intermediate aluminum carbide is produced. High-temperature aluminum carbide reacts instantly with water to produce copious quantities of highly inflammable methane gas, which is the common natural gas combustion fuel that can even be explosive. The spontaneous combustion temperature of methane is only 570 °C. Temperatures around Grenfell Tower would reach an excess of 2000 °C. The otherwise slower burning aluminum–polymer cladding needed nothing more than cold water to turn what was a domestic fire, manageable by smothering containment, and evacuation, into the catastrophic inferno that was the Grenfell disaster (Figure 1). Indeed, if water had not been used, it seems likely that the blaze would not have burned so hot nor spread so quickly, leaving time for more efficient evacuation as was the case with the Address Downtown Dubai fire (vide infra).

Many reports in the media have already suggested that the cladding used for the Grenfell Tower refurbishment contributed to the rapid spread of fire. Online YouTube film footage shows flames shooting up the side of the building and sheets of flaming material ‘raining’ down. There has been a great deal of speculation as to why the fire spread so rapidly: the fire broke out on a warm night; windows were open with curtains blowing in the breeze, which would have contributed to the rapid spread of fire within the building; there were chimney ventilation effects from the cavities, etc. Notwithstanding all of these observations, the basic reason that the fire spread out of control in just a few minutes is that water was sprayed upon a combination of burning molten plastic and aluminum.

Notice that the flux of water hitting the lower floors is quite weak and insufficient to cause any deluge effect. When the water hits the lower levels, it reacts with Al_4_C_3_ and Al to form large amounts of methane and hydrogen. This gas ignites in a vertical wall of flame that rapidly rises and ignites the higher levels. Since the gases also contain hot water vapor, there is positive feedback that leads a rapid, mostly vertical, facade configuration that is a Hallmark of such events.

In the following sections we review (1) a brief survey of previous similar disasters, with lessons that evidently have not yet been properly learned and instigated by the fire-fighting authorities; (2) the properties of the combustible construction materials; (3) the thermochemical reactions involved; and (4) the evidence that the water caused the acceleration of the conflagration from the timeline of events. In the appendix, we review evidence that the 9/11 collapse of the World Trade Center twin towers may also have been caused by similar reactions and cite other instances where the fires involved buildings that were known to have been refurbished with aluminum panels.

## 2. High-Profile Aluminum Fires

There have been several previous high-profile tower-block fires, which are all associated with the use of water as an ‘extinguisher’ on aluminum–polymer clad buildings, although still not recognized as the cause of rapid conflagration by the various authorities involved.

### 2.1. Garnock Court, Ayrshire (1999)

This was the tower-block fire that first raised concerns over polymer and aluminum cladding on high-rise buildings. Witnesses told how the flames leapt up yellow-colored cladding on the corners of the block, taking just five minutes to spread to the top. The blaze prompted a parliamentary inquiry into tower-block cladding, which recommended a much tougher testing regime, and the use of non-combustible materials. One witness reported, “There were parts of the cladding dropping on to the first appliance that “could not be moved as it was supplying the fire fighters with water.” A subsequent Parliamentary enquiry concluded that when fire spreads externally via the cladding, guidance for this type of fire might not be adequate to prevent the conflagration. It further concluded that cladding systems should be required either to be entirely non-combustible, or to be proven through full-scale testing not to pose an unacceptable level of risk in terms of fire spread. 

### 2.2. Lakanal House Fire (2011)

Six people died in this Southwark London tower block fire, and many were injured. The fire started as an electrical fault. A coroner’s report in 2013 found problems with fire safety including the buildings’ fire resistance. The London Fire Brigade also opened an investigation into the fire; the report revealed that Lakanal House had been identified as being at risk of enabling a fire to spread if one should occur in one of the flats. Although it was originally reported that some of the windows made of plastic (ultra-high-density polyvinyl chloride), windows in the block were in fact made of aluminum. An inquest into the deaths at Lakanal House “found the fire spread unexpectedly fast, both laterally and vertically, trapping people in apartments, with the exterior cladding panels burning through in just four and a half minutes”. As in the case of the Grenfell Tower fire six years later, the official advice was for people to remain in their homes in the event of a blaze. The inquest concluded that years of “botched renovations” had removed fire-preventative material between flats and communal corridors, allowing a blaze to spread, and that the risk of rapid conflagration was not identified in any of the safety inspections. 

### 2.3. Lacrosse Tower in Melbourne (2014)

The Lacrosse multi-story apartment block caught fire in November 2014 in the dockland area of Melbourne, Australia, resulting in a rapid conflagration in a similar manner to London’s Grenfell Tower. It was believed to have started when a cigarette burning on an eighth-floor balcony of the residential tower sparked a fire that raced up the aluminum-clad walls to the 21st floor within 11 min. A post-incident report said aluminum composite panels that were not approved for external use on a high-rise building in Australia were the direct cause of the “speed and intensity of the fire spread”. A closer investigation of the pictures and timeline of events bear a remarkable similarity to the Grenfell Tower disaster. This suggests that the Lacrosse fire also erupted when water was first applied to extinguish the embryonic flames.

### 2.4. Dubai Fires (2012–2016)

There have been five well-publicized aluminum cladding fires in the city of Dubai since 2012. Dubai is renowned for its shiny rocket shaped skyscraper tower blocks that characterize the city skyline. The first aluminum cladding fire was in 2012, when the 40-story Al Tayer Tower residential block erupted in flames, and later the same year, another residential block, the 37-story Tamweed Tower, went up in flames. In another Dubai fire Figure 2 in March 2015 known as the Torch Tower blaze, a 79-story residential and office block also quickly and dramatically went up in flames. Fortunately, just before the Torch Tower fire, the authorities had put in place a protected access and evacuation system, so that the fire fighters were able to use this safety lift to get rescue forces up to the area of the fire and safely evacuate all the occupants. The latest Dubai fire was a 75-story residential tower just one year before Grenfell in July 2016.

However, the most dramatic of all the Dubai tower-block fires was on New Year’s Eve 2015 at the Address Downtown Dubai Hotel, which stand’s adjacent to Dubai’s tallest skyscraper and indeed also the world’s tallest building: the 850 m high Burg Khalifa. Dubai’s relatively modern tower blocks are all fitted with sprinkler systems to protect apartment fires from escalating and to allow evacuation. The Address Hotel fire coincided with the beginning of the New Year’s Eve fireworks celebrations from the Burj Khalifa super-tower. According to Dubai Civil Defense record of events, sprinkler systems in the fire at the Address Downtown Dubai Hotel *ran out of water* 15 min into the breakout of fire.

Was this a blessing, we ask? The extent of the Dubai Address Hotel blaze was beyond the capacity of regular sprinkler systems to cope with; it was mainly an external fire across more than 40 floors. Compared to Grenfell, the fire spread was relatively slow, with no fatalities. Everyone was evacuated, leaving just 15 people with minor injuries, and one person suffered a heart attack. When the firefighters reached the Address Downtown Hotel, they were swiftly able to clear 3000 people.

Perhaps the reason for the damage-limited conflagration, and successful evacuation, can be explained to some degree by the fact that, owing to pressure on the fire safety systems that day, the Dubai Address Hotel tower building sprinkler system exhausted its water supply within 15 min of the start of the fire. There was also no water available for the fire fighters’ hoses! Putting water sprinklers in aluminum-clad tower blocks could exacerbate the risk of non-survival, in similar conflagrations to Grenfell, rather than offer any more protection to residents. 

## 3. “ALICE” NASA Rocket Fuel

Interestingly, NASA scientists recently test-launched a rocket with a new fuel propellant formulated by mixing aluminum powder and water (powdered ice), which is called ‘ALICE’ [3]. Thermochemical engineers have known for years that aluminum reacts exothermically with water, giving off hydrogen gas plus heat energy. While solid aluminum requires excess heat to ignite the reaction with water, nano-aluminum has a much greater surface area and will react with water at around 650 °C, i.e., as the aluminum begins to melt. At this temperature, the nano-aluminum with water can be ignited with a small flame. The same applies to molten aluminum when sprayed with water.

The US Defense Agencies have applied considerable resources to research fire hazards. While some of that work is classified, much is available in the open-access scientific literature. For example, Zabel’s group at the Southwest Research Institute at San Antonio, Texas has compiled a comprehensive military handbook [4] that addresses, inter alia, the kind of special particulate-loaded fire extinguishers needed to fight aluminum fires. The US Air Force uses Al_2_O_3_ powder-based extinguishers. It has also been documented how various aluminum composite (thermite) formulations can be used to very effectively burn through the armor plating of combat vehicles [5]. The special hazards of aluminum-based fires are well-known to the aerospace research community. However, this information needs to more widely disseminated throughout the civilian fire-fighting community. 

## 4. Cladding and Roof Materials

High-rise buildings are designed to contain fires within the flat where they may break out. The basic reason why people are told to stay put inside their flats is that there is a presumption that the building construction materials and the application of water hoses, if need be, will prevent the fire from spreading. The original Grenfell cladding when the building was constructed in 1970 was precast ceramic panels to window height with single-glazed aluminum-framed windows above. The thermal insulation of this type of façade is poor, but the fire resistance is good. 

**Figure 3:**
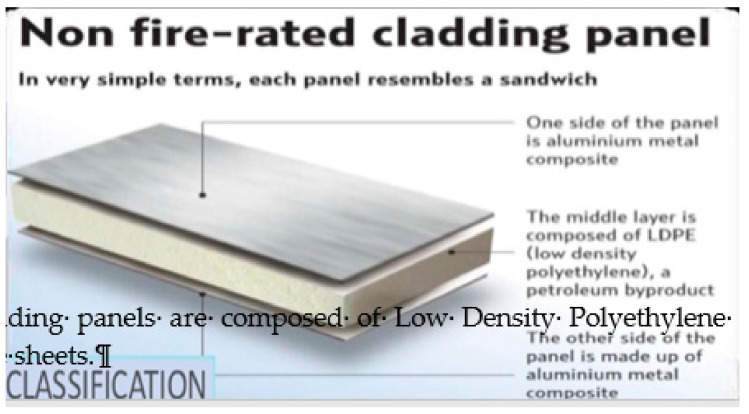
Aluminum siding panels are composed of low-density polyethylene (LDPE) sandwiched between aluminum metal composite sheets.

The Grenfell Tower was upgraded using funding from the ECO (Energy Company Obligation), which is a UK-government energy efficiency program. The cladding thermal insulation Figure 3 was a ‘Reynobond Celotex RS5000′ insulation panel and cladding. Its core material is mainly polyethylene. There was a 50 mm gap between the insulation and cladding. The cladding was available in two variants. The plastic construction choice enables a rigid yet lightweight panel, but it poses a greater fire hazard. 

The facade windows in Grenfell Tower window frames were made from polyester (PMMA– polymethyl methacrylate) powder coated aluminum. 

ACM (aluminum composite material) cladding consists of two panels of aluminum bonded to either side of a lightweight core of an insulating material such as polyethylene (PE). The insulation plastic was a thick layer, 100 mm to 150 mm. The aluminum rain screen is fitted over the cladding insulator panels to protect the insulation from the weather and provide a decorative finish. This is separated from the insulation by a 50 mm wide cavity. Thermal insulation, air-tightness, and structural stability are provided by the inner part of the wall construction. 

Since polyethylene-cored plastic foam cladding panels were used alongside aluminum sheets, and with a few millimeters of air in between, it is hardly surprising that external fires take hold quickly. The aluminum facing is resistant to the surface spread of the flame, but this would be of little use where the intensity of the fire would quickly melt the thin aluminum, which has a relatively low melting point (660 °C). The outer aluminum panel would be cooler than the building wall when the fire starts, so the molten plastic would preferably stick to the aluminum, creating favorable conditions for the production of Al_4_C_3_. Cavities in buildings can contribute to the spread of fire, as these function similar to a chimney, drawing flames upwards. Thus, once fire takes hold, all that is needed to spread a firestorm upwards and out of control, with the liberation of flammable gases hydrogen and methane, is a relatively small amount of water. Copious amounts of hot hydrogen and methane gases liberated when water hits Al or Al_4_C_3_ go upwards by convection and thus rapidly accelerate the propagation of fire upwards by bringing live flames to new quantities of plastic and aluminum. In effect, water added to such fires is literally “adding fuel to the flames”. 

## 5. Combustion Chemical Reactions

### 5.1. Combustion of Plastics

**Figure 4:**
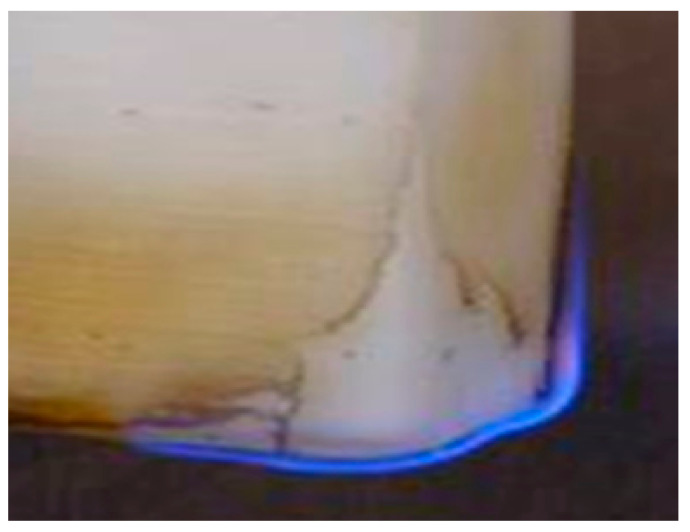
Initial stages of burning a sample of polyethylene (PE). Note: Solid PE burns only after it has been melted by a contiguous flame.

Polyethylene gets hot slowly as its specific heat is relatively high (1900 J.°K−1 kg−1) and its thermal conductivity is relatively low (0.45 to 0.52 W.m^−1^ °K^−1^). Figure 4 illustrates how solid polyethylene burns with a slow and steady blue flame. The flash point of high-density polyethylene is 340 °C; the autoignition point is 380 °C. The flame spreads slowly along the surface, melting the solid polyethylene to liquid as it spreads. The melting point of polyethylene is 100 °C to 135 °C, depending on the density. Molten polyethylene in the laboratory (Figure 4) drips flaming drops, but in a contained environment—for example, sandwiched between aluminum sheets, with an air cavity—the whole sample would quickly become liquid. Solid PE burns only after it has been melted at the surface by a contiguous flame; it is the vapor at the surface of the molten plastic that combines with the oxygen of the air during combustion; the rate of combustion can accelerate if the surface area of a molten plastic is dispersed, for example, by the addition of water. When bulk polyethylene burns rapidly with a limited supply of oxygen, the combustion produces clouds of black carbon aerosol fumes. Any containment or fire control system should allow for the low melting point of polyethylene. The following combustion reaction occurs when PE solid melts, and then liquid, polyethylene burns in air, with limited oxygen diffusion to the molten polymer/air interface, i.e., at the flame.

Polyethylene + oxygen (air)→carbon + water X (C_2_H_4_)n (solid) + Xn O_2_ (gas) → 2nC(aerosol) + 2nH_2_O (vapor) ΔH =−13.1 kJ/gO_2_ (1)

The combustion product, carbon, can take the form of both a solid residue and a black aerosol smoke. Some of the carbon burns to carbon dioxide (CO_2_) and carbon monoxide (CO) gases. Flame temperatures are estimated to be between 1000 and 1500 °C. The heat of combustion is taken from Walters and Hackett [6]. Their experimental results were compared with thermochemical calculations of the net heat of combustion from oxygen consumption and the gross heat of combustion from the group additivity of the heats from the formation of products and reactants. The gross and net heats of combustion calculated from polymer enthalpies of formation and oxygen consumption thermochemistry were all found to be within 5% of the experimental values from oxygen bomb calorimetry. The net heat released by combustion per gram of oxygen consumed is 13.1 ± 0.8 kJ/gO_2_ for all polymers tested, including polyethylene. Using this average result, and chemical Equation (1), we very roughly estimate that a kilogram of burned polyethylene yields an amount of heat of 1000 × 13.1 × 8/7 = 15 MJ/ kg, where 8/7 is the molecular weight ratio of oxygen to ethylene (32/28).

### 5.2. Reaction of Molten Aluminum with Water

The melting temperature of aluminum at 1 atm with a latent heat of fusion (ΔH_f_) is:

660 °C (1 atm.) Al (solid) → Al (liquid) ΔH_f_ = − 387kJ/kg (2)

nd the boiling point of liquid aluminum is 2470 °C. We do not know that the fire reaches this high temperature, but it may be possible.

The mean heat capacity (C_p_) of solid aluminum over its range of existence from say 25 °C to 660 °C at 1 atm is < C_p_ > = 0.96 kJ/(kg.°C). Thus, we can calculate that the total energy required to heat, and then to melt, 1 kg of aluminum is 635 × 0.96 + 387 = 997 kJ of heat. This amount of heat is produced in the combustion of around only 67 g of polyethylene!

It is well known in the energy industry that over the entire temperature range of its existence, aluminum reacts spontaneously with water to produce hydrogen [7]. The reaction at all temperatures is exothermic with a very large heat of reaction (see Table 1).

The first reaction forms the aluminum hydroxide bayerite (Al(OH)_3_) and hydrogen, the second reaction forms the aluminum hydroxide boehmite (AlO(OH)) and hydrogen, and the third reaction forms aluminum oxide and hydrogen Equation (3).

2Al + 3H_2_O (liquid) → Al_2_O_3_ (solid) + 3H_2_(gas) ΔH_1000_ = –366 kJ/molH_2_ (3)

All these reactions are thermodynamically favorable from room temperatures upwards. All are also highly exothermic. From room temperature to 280 °C, Al(OH)_3_ is the most stable product, while from 280–480 °C, AlO(OH) is most stable. Above 480 °C, alumina Al_2_O_3_ is the most stable product; meanwhile, Al_2_O_3_ becomes increasingly more thermodynamically favorable than the hydroxide Al(OH)_3_ at elevated temperatures. 

The thermodynamic parameters for Al_2_O_3_ reactions are reproduced from reference [6] in Table 1 at three temperatures up to 1000 °C. The tabulated values are per mol H_2_ produced. The enthalpy (ΔH) is highly exothermic at all temperatures, with a very high value of –366 kJ/mol H_2_ at 1000 °C. Over this temperature range, the entropy change (ΔS) goes from positive to negative, reflecting that water becomes steam above 100 °C. The Gibbs free energy of the reaction (ΔG) decreases sharply as temperature increases, thereby driving the reaction rate faster. The hydrogen that is liberated will spontaneously burn in the air to water plus additional large amounts of energy. For example, at 1000 °C, the heat of combustion of hydrogen is of the order 1 MJ per mole of oxygen.

2H_2_ (gas) + O_2_ (gas) → 2H_2_O (steam) ΔH = + ~ 1 MJ/mol O_2_ (4)

‘ALICE’ is an acronym for a fuel used in the aerospace industry comprising a mixture of aluminum powder and water, which is in the form of ice for safe storage. The mixture reacts by Equations (2) and (3) to produce huge amounts of energy. It is used by NASA as a rocket launch propellant when burned at high temperatures around 1000 °C [3].

### 5.3. Formation of Aluminium Carbide

**Figure 5:**
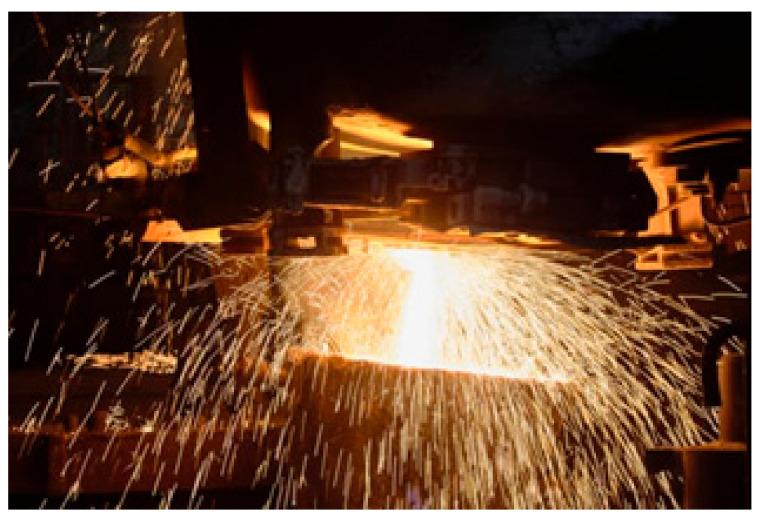
Interation of vaporized aluminum with carbon at a temperature of 1800 °C in an electric furnace produces aluminum carbide (Al_4_C_3_).

The heat of formation of Al_4_C_3_ at around 1330 °C is a massive 1275 KJ per mole [8]; the enormous amount of energy that is released when Al_4_C_3_ is formed can be compared with the heat of formation of water vapor at 1 atmosphere pressure from the explosive reaction of hydrogen with oxygen, which is 241.8 KJ per mole of water. The heat of formation of AL_4_C_3_ is so great that it takes the form of white hot liquid sparks similar to fireworks (Figure 5).

### 5.4. Hydrolysis of Aluminum Carbide

Hydrolysis is a reaction that takes place between a substance and water, as a result of which the substance and water break down, and new compounds form. Al_3_C_4_ is a salt-like carbide, which is essentially the product of the displacement of all the hydrogen atom in methane by Al atoms. During hydrolysis, reverse displacement takes place easily, and methane forms. The hydrolysis of aluminum carbide is a spontaneous irreversible reaction with a very large negative Gibbs free energy of reaction at all temperatures. This reaction is often used as a simple room temperature method of obtaining methane in the laboratory; the hydrolysis of Al_4_C_3_ at low temperatures gives aluminum hydroxide.

2H_2_ (gas) + O_2_ (gas) → 2H_2_O (steam) ΔH = + ~ 1 MJ/mol O_2_ (5)

whereas at the high combustion temperatures above 1000 °C, the solid product is alumina.

2H_2_ (gas) + O_2_ (gas) → 2H_2_O (steam) ΔH = + ~ 1 MJ/mol O_2_ (6)

### 5.5. Combustion of Hydrogen and Methane

The combustion reaction of hydrogen with oxygen is highly exothermic with a heat liberated of 572 kJ per mole of hydrogen.

2 H_2_(gas) + O_2_(air) → 2 H_2_O (liquid-mist) ΔH_300_ = + 572 kJ/molH_2_ (7)

Hydrogen gas forms explosive mixtures with air in concentrations from 4%–74%; although the spontaneous combustion temperature (585 °C) is higher than some hydrocarbon fuels, such as methane, it is much more dangerous as the auto-ignition energy barrier is extremely small; explosive reactions may be triggered by a spark, simply low heat, sunlight, or any hot metal oxide.

The auto-ignition or spontaneous combustion temperature of methane in the presence of oxygen–air is 537 °C, so the CH_4_ then further fuels the conflagration with the exothermic combustion of the fuel gaseous methane, which can also be explosive in its reaction with oxygen under certain conditions.

**Figure 6:**
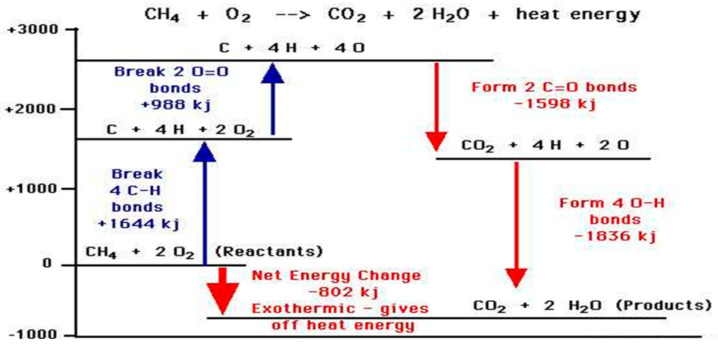
The heat of the combustion energy of methane obtained from standard tables of bond energies explains how it originates; 1 kg of aluminum can combine with carbon and water to produce 30 moles of CH_4_, which reacts with oxygen in air to liberate to the environment 0.8 × 30 = 24 megajoules of heat.

Figure 6 explains how amounts of reaction heat can be approximately estimated from bond energy tables; the stronger the chemical bonds of the product, such as water and CO_2_, the greater the release of energy on combustion.

### 5.6. Overall Reactions

C (solid) + O_2_ (air) → CO_2_ (gas) 2Al (liquid) + 3H_2_O (liquid) → Al_2_O_3_ (solid/aerosol) + 3H2 (gas) 4Al (liquid) + 3C (solid/aerosol) → Al_4_C_3_ (solid/aerosol) Al_4_C_3_ (solid/aerosol) + 6H_2_O (liquid) → 2Al_2_O_3_ (solid/aerosol) + 3CH_4_ (gas) 2H_2_ (gas) + O_2_ (air)→ 2H_2_O (steam) CH_4_ (gas) + 2O_2_ (air) → CO_2_ (gas) + 2H_2_O (steam) (8)

An estimate of the heat balance with excess water, and oxygen surface-limited combustion of polyethylene to melt the aluminum, for the overall combustion of a mixture of aluminum and water is of the order + 20 megajoules of energy liberated per kg of aluminum at around 1000 °C. This massive energy drives the very high-temperature fire.

## 6. Conclusions

The interior of flames from burning molten plastic can reach temperatures between 1000 °C and 1500 °C, giving off a black smoke which is a carbon aerosol, and black carbon residues. At temperatures at or near burning molten plastics, aluminum becomes molten (above around 700 °C), highly fluid, highly volatile, and highly reactive. The aluminum can react directly with carbon aerosols and residues to produce aluminum carbide. The heat of formation of Al_4_C_3_ is so great that it appears transiently as white-hot liquid sparks, which is similar to magnesium oxide in fireworks, as reportedly seen by the firefighters and observers nearby.

When water is added to aluminum carbide, methane is produced along with alumina dust and steam and carbon dioxide, which is also a white smoke. When water is sprayed onto hot molten aluminum, hydrogen gas is produced along with alumina as a white dust and steam or water vapor. All the thermodynamic data and analysis shows that both these reactions would have played a major role in the rapid spread of the conflagration. Without more forensic evidence than available here, we are unable to say with any certainty to what extent the aluminum burned directly with water via reaction Equation (3) producing hydrogen, or reaction Equation (6) producing methane. However, whichever of these two reaction mechanisms prevailed, the result would be the same; a rapid catastrophic produced a mostly vertical spread of a façade conflagration, as both H_2_ and CH_4_ are highly inflammable—and under certain conditions, explosive—gaseous fuels. The Gibbs energy changes for both these reactions are extremely large and negative; thus, both reactions will undoubtedly occur if the reactants are present. Moreover, the overall products of aluminum combustion with water are gases that are removed rapidly from the reaction mixture by convective forces, thus driving all reaction pathways to rapid completion. 

Therefore, we conclude that the application of water to extinguish aluminum fires is worse than futile; it accelerates the conflagration and can be explosive. This is the scientific explanation of the ferocity and speed of the spread of Grenfell Tower fire, which caused it to reach the top of the building in less than 15 min and eventually encircle the entire building whilst water was continuously being directed onto the flaming building from various directions. 

In conclusion, the thermodynamic analysis presented here highlights the massive thermal energy that drives aluminum fires. The presence of carbonaceous matter in close proximity to aluminum provides a pathway for the formation of aluminum carbide. Spraying water at this stage liberates methane and—at higher temperatures—hydrogen gas. We note that the underlying chemical thermodynamics provides a very plausible mechanism that is in accord with the observations namely:

- Very high combustion temperature
- Rapid spread in the vertical direction
- Characteristic smoke emissions
- Large “sparks” emitted
- Reported explosions.

Clearly, it would be desirable to establish, as a matter of some urgency, the detailed mechanism of initiation and propagation. It would be straightforward to analyze the reaction products that should contain copious amounts of aluminum oxide, a long-lived refractory oxide, which might be expected to deposit in the direction of the plume along with particulate carbon. The particle sizes and composition will provide evidence of their origin. Highly instrumented laboratory tests, using Raman scattering techniques to analyze the in situ composition of the fire plume would also be useful. Finally, it is clearly essential that fire testing standards need to be developed for this class of building material so that the effects of applying water (or sprinklers) may be better understood. It would seem particularly unhelpful to install water-based sprinkler systems in buildings that contain or are coated with large amounts of aluminum. Aluminum has many advantages in building application so that a better understanding of how to formulate composite panels that retain the advantages of aluminum while substantially mitigating the fire risk could be developed.

It is clearly imperative that the senior scientists who are responsible for developing, implementing, and enforcing standards ought to be better educated in the scientific fundamentals so that they might recognize that what might seem to be innocuous changes may introduce represent huge fire risks; fire fighters on the ground need to be made aware of building classifications in their areas so as to implement effective fire-fighting strategies. This will require improved training to the level of “boots on the ground”.

**Figure 7:**
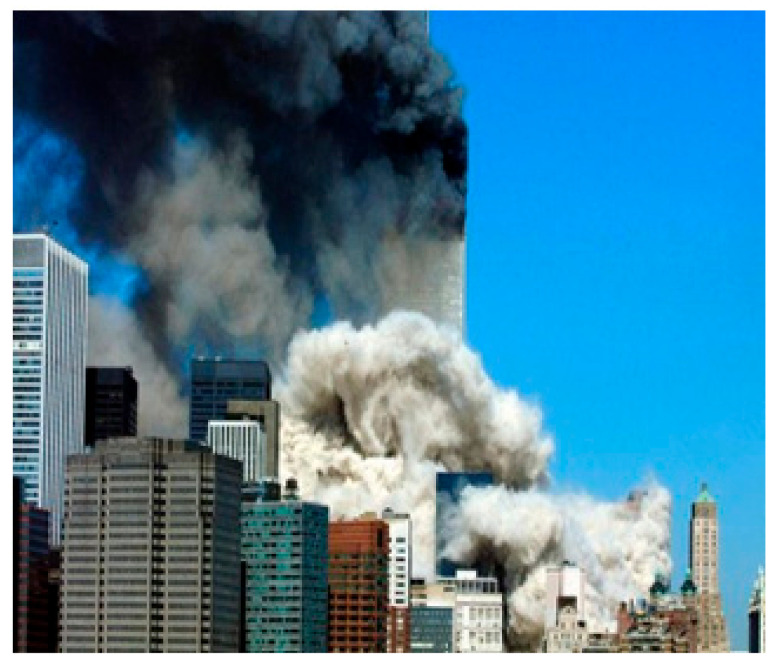
Collapse of New York World Trade Center 11 September 2001.

In support of the conclusion of this work, we cite the thermochemical explanation of the world’s greatest ever tower-block disaster: the collapse of the New York World Trade Centre twin towers on 11 September 2001 after being hit by aeroplanes (Figure 7). The fundamental reason for the very intensive fire that led to the total collapse was inexplicable at the time of the subsequent official forensic investigations and for several years after. Senior scientist Christian Simensen of SINTEF Materials and Chemistry (Norway) has presented the only plausible scientific explanation of what really caused the collapse of the twin towers when they were attacked by the aircraft, at an international materials technology conference [9]. 

Black and white smoke billowed up as the first of the two WTC towers collapsed; the burning kerosene fuel and burning plastic is characterized by black smoke (second WTC: carbon aerosol and water vapor), while the burning of aluminum and water reaction gases is characterized by white smoke (first WTC: alumina dust and water vapor). Hydrogen liberated in the Al + water reaction ‘exploded’ as the first twin tower collapsed amidst plumes of white smoke. 

When the aircraft became jammed inside layers of building debris, the mainly aluminum bodies rather than the buildings themselves absorbed most of the heat from the burning aircraft fuel. This vast amount of heat would melt approximately all the 30 tons of aluminum in the aircraft fuselage and increase the molten aluminum temperature. The fluidity, i.e., reciprocal viscosity, of liquids increases exponentially with temperature; molten aluminum above 1000 °C is more fluid than water, and it is also highly volatile. The aluminum poured downwards within the tower blocks through staircases and gaps in the floor, undergoing the chemical reaction with water from sprinklers. All floors of the twin towers’ 80 stories were equipped with an automatic water sprinkler system, which was triggered by rise in temperature. A mix of the sprinkler system water and hot molten aluminum and aluminum carbide formed from the combustion of the cabin interior and office furniture reacted to produce hydrogen and methane. This caused the rapid and powerful spread of the high-temperature fire vertically. When the hydrogen/methane mixture reached explosive limits, a number of loud and powerful explosions occurred, which were heard by both firefighters and many witnesses at the scene. This combination of high temperatures that weakened the supporting beams and powerful explosions led to the collapse of the upper floors and rapidly to the total collapse of the building in accord with well-accepted principles of mechanical failure analysis.

**Figure 8:**
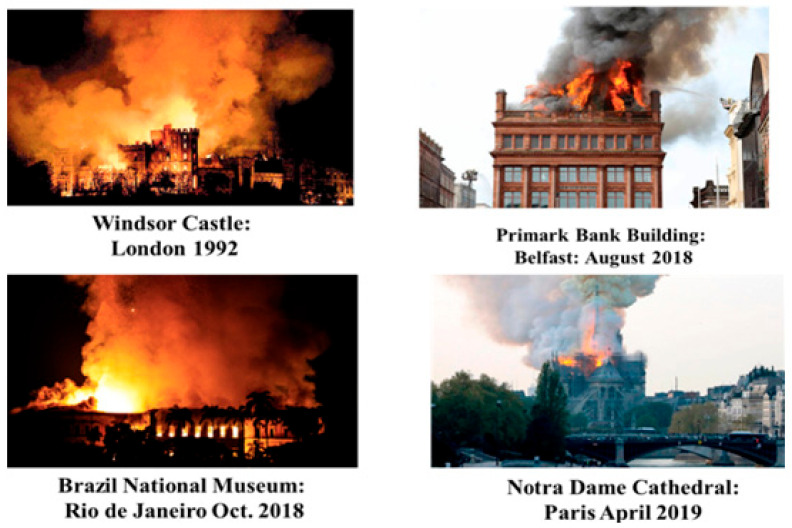
Catastrophic building fires that occured after or during roof refurbishment where aluminum paneling may have played a role.

Finally, we mention that there have also been several high-profile fires involving buildings known to have been refurbished with aluminum roof panels (Figure 8). In all these cases, there is the evidence from video footage, which is still available on YouTube, of accelerated conflagration and white smoke when the water is applied as an extinguisher. It is essential that the fire testing of these aluminum and composite aluminum plastic materials include the spraying of water on the molten burning research samples.

## Figures and Tables

**Figure 2:**
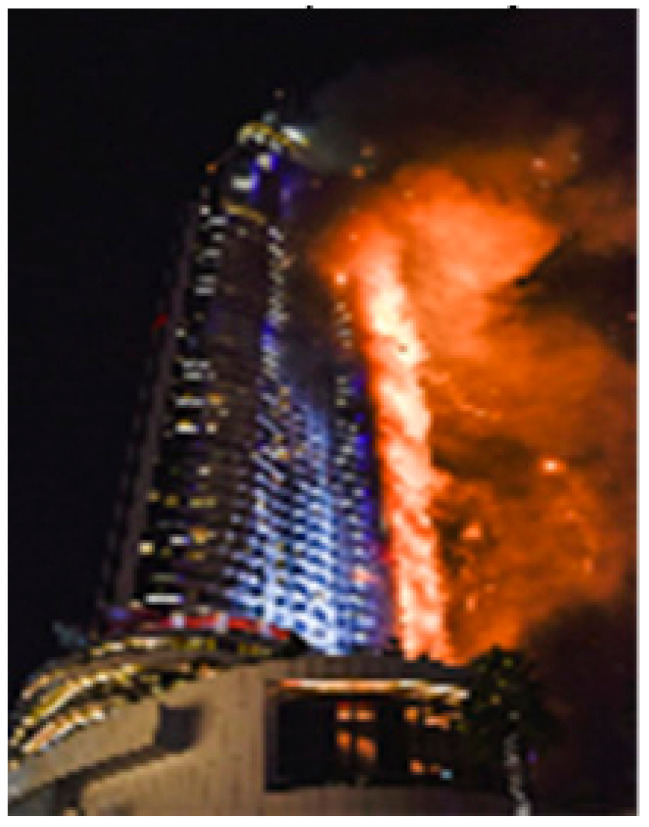
Dubai’s Address Downtown Hotel, New Year Celebration fire, 31 December 2015.

**Table 1:** Thermodynamic data for the aluminum–water reaction to form alumina.

T (°C)	ΔH kJ/mol H_2_	ΔS J/K	ΔG kJ/molH_2_
0	−272	62.1	−289
100	−275	51.1	−294
1000	−366	−51.6	−304
